# Community knowledge and practices regarding antibiotic use in rural Mozambique: where is the starting point for prevention of antibiotic resistance?

**DOI:** 10.1186/s12889-020-09243-x

**Published:** 2020-07-29

**Authors:** Olga Cambaco, Yara Alonso Menendez, John Kinsman, Betuel Sigaúque, Heiman Wertheim, Nga Do, Margaret Gyapong, Johannes John-Langba, Esperança Sevene, Khátia Munguambe

**Affiliations:** 1 Centro de Investigação em Saúde de Manhiça (CISM), Manhiça, Mozambique; 2 Department of Epidemiology and Global Health, Umeå University, Umeå, Sweden; 3 Department of Medical Microbiology and Center for Infectious Diseases, Radboudumc, Nijmegen, Netherlands; 4 Oxford University Clinical Research Unit, Hanoi, Vietnam; 5 Centre for Health Policy and Implementation Research, Kintampo, Ghana; 6 University of Kwazulu-Natal, Durban, South Africa; 7 Faculdade de Medicina, Universidade Eduardo Mondlane, Maputo, Mozambique

**Keywords:** Antibiotics, Antimicrobial resistance, Community, Awareness, Mozambique

## Abstract

**Background:**

Antibiotic misuse and other types of unnecessary use of antibiotics can contribute to accelerate the process of antibiotic resistance, which is considered a global concern, mostly affecting low-and middle-income countries (LMICs). In Mozambique there is limited evidence on community knowledge and practices regarding antibiotics and antibiotic resistance. As part of the ABACUS project, this paper describes knowledge and practices of antibiotic use among the general population in the semi-rural district of Manhiça to inform evidence-based communication intervention strategies for safer antibiotic use.

**Methods:**

The study was conducted in Manhiça, a semi-rural district of Southern Mozambique. Sixteen in-depth interviews and four focus group discussions (FGDs) were conducted with community members to explore lay knowledge and practices regarding antibiotics and awareness of antibiotic resistance. The qualitative data was analysed using a combination of content and thematic analysis. The SRQR guidelines for reporting qualitative studies was performed.

**Results:**

Although participants did not hold any consistent knowledge of antibiotics, their visual recognition of amoxicillin (distinct red yellow capsule) was acceptable, but less so for different types and brands of antibiotics. The majority of participants were aware of the term ‘antibiotic’, yet the definition they gave was rarely backed by biomedical knowledge. Participants associated antibiotics with certain colours, shapes and health conditions. Participants reported common habits that may contribute to resistance: not buying the full course, self-medication, sharing medicines and interruption of treatment. Most had never heard of the term ‘antibiotic resistance’ but were familiar with the phenomenon. They often understood the term ‘resistance’ as treatment failure and likened ‘resistance’ to non-compliance, ineffective medication, disease resistance or to an inability of the physical body to respond to it.

**Conclusion:**

There is a broad understanding of the importance of medication compliance but not specifically of antibiotic resistance. In addition, there is a recognized gap between knowledge of responsible drug compliance and actual behaviour. Future qualitative research is required to further explore what determines this behaviour. The existing ability to visually identify amoxicillin by its distinct red and yellow appearance is informative for future awareness and behavioural change campaigns that may incorporate visual aids of antibiotics.

## Background

Antimicrobial resistance (AMR) is considered a global public health problem particularly affecting low- and middle-income countries (LMICs) [1, 2]. The World Health Organization (WHO) has long recognized AMR (and specifically antibiotic resistance) as a growing global health threat and considers it a high priority [3]. A driving force behind the rise in antibiotic resistance in LMICs is their “inappropriate” use. This phenomenon can be understood through factors on the supply side, owing to the practice of excessive prescription, uncontrolled or uneven access and, on the demand side, to the use of antibiotics [4–7].

The factors determining use of antibiotics from the demand side are influenced by several aspects, including: consumers’ lack of knowledge about appropriate antibiotic use and its implications, as well as beliefs, expectations and personal experiences with antibiotics [6, 8–17].

Although the evidence of absolute consumption of antibiotics is most numerous in high-income regions, such as Europe, antibiotic consumption is growing the fastest in LMICs [18]. In addition, since antibiotics can be purchased without prescription in many LMIC settings, self-medication is a common practice among consumers [8, 10–12]. A recent public awareness survey conducted in selected regions of all continents, including sub-Saharan Africa, south-east Asia and central America, revealed misunderstandings about conditions treatable with antibiotics and the concept of antibiotic resistance [3], whilst a narrative review in Europe found that lack of public knowledge and awareness one of major factors among the general public driving antibiotic resistance [18].whilst other studies confirmed that community members lack ed. knowledge to differentiate between antibiotics and other commonly used medicines [9, 12, 19–24].

In the case of Mozambique, besides the inexistence of specific antibiotic regulation, the “Medicines law” [25] defines the list of over-the-counter drugs that must be updated on a regular basis. Antibiotics are not included in this list [26], meaning that health care providers are not allowed to dispense antibiotics without a prescription. However, a recent study reported that antibiotics comprised 20% of the most frequently purchased drugs by consumers with approximately 54% of customers purchasing the drugs without a prescription [27]. In spite of what is legally required, a recent study conducted in Maputo, concluded that the proportion of use of non-prescribed antibiotics was higher in those who purchased from informal markets (82.6%) [28]. Another study conducted in the same area and focusing in informal vendors, concluded that the informal market in Mozambique poses a serious public health risk considering that the vast majority of informal drug dispensers (82%) have no pharmaceutical training [27]. The first official situation analysis on antibiotic use and resistance in Mozambique suggests that there are likely high levels of inappropriate antibiotic use at the community level and it recommends the rationalisation of antibiotic use in the community as one of the strategies to reduce antibiotic resistance [29]. Two recent studies conducted in Mozambique confirmed both the existence of inappropriate use of antibiotics at the community level and the prevalence of self-medication via the purchase of non-prescribed antibiotics [28, 30]. Additionally, both studies conducted in urban areas revealed the limited knowledge of antibiotic use that the study population held.

The limited evidence presented thus far suggests that there is much to be done with regards to understanding and addressing both the supply and demand-driven factors that determine inappropriate antibiotic use, yet it is precisely in such contexts where regulations are not enforced that the knowledge of the general public (the demand side) can play an important role in the reduction of inappropriate use of antibiotics [9, 19, 31].

Awareness studies that evaluate lay knowledge of antibiotics and the factors underlying the unsafe use of antibiotics are considered crucial in order to inform efforts to improve community understanding and best practices in low-resource countries [31]. Knowledge, attitudes and practice (KAP) studies are often a preferred method to achieve this [6, 15, 17, 32–35]. These KAP studies focus on the appropriate consumption, attitudes towards, and misconceptions of antibiotics, but do not conduct in-depth examinations of antibiotic knowledge (including visual recognition) and tend to be methodologically limited to categorical survey data.

There is limited qualitative research published to date regarding community knowledge of antibiotics or antibiotic resistance and practices regarding antibiotic use in Mozambique, especially with regards to rural areas. This study was designed to address this knowledge gap. The objective of the study was to use a bottom-up approach to describe different dimensions of community knowledge of antibiotics and practices regarding antibiotic use in Mozambique from the perspective of community members so as to provide much-needed empirical evidence for message development and positioning in the context of information, education and communication (IEC) strategies to improve appropriate use of antibiotics.

## Methods

### Study design

This paper is based on analysis drawn from an observational mixed-methods multi-country study – the ABACUS study (Antibiotic Access and Use). ABACUS investigated ‘community-level antibiotic access and use in 6 LMICs (Bangladesh, Mozambique, Vietnam, Ghana, Thailand and South Africa) in order to inform the design of, and identify targets for, community-based interventions serving to promote appropriate antibiotic use [36]. The quantitative phase of the study consisted of a community mapping exercise coupled with an inventory of antibiotic suppliers using a census approach, exit interviews with clients of antibiotic suppliers and community-based cross-sectional household questionnaires. The qualitative phase of the ABACUS study in Mozambique consisted of in-depth interviews and focus group discussions conducted with community members and antibiotic suppliers, conducted from March 2016 to May 2018. The results and analysis presented in this article were drawn from the qualitative component of the Mozambican study site, although focusing only on the community members target group.

### Study site

**Fig. 1:**
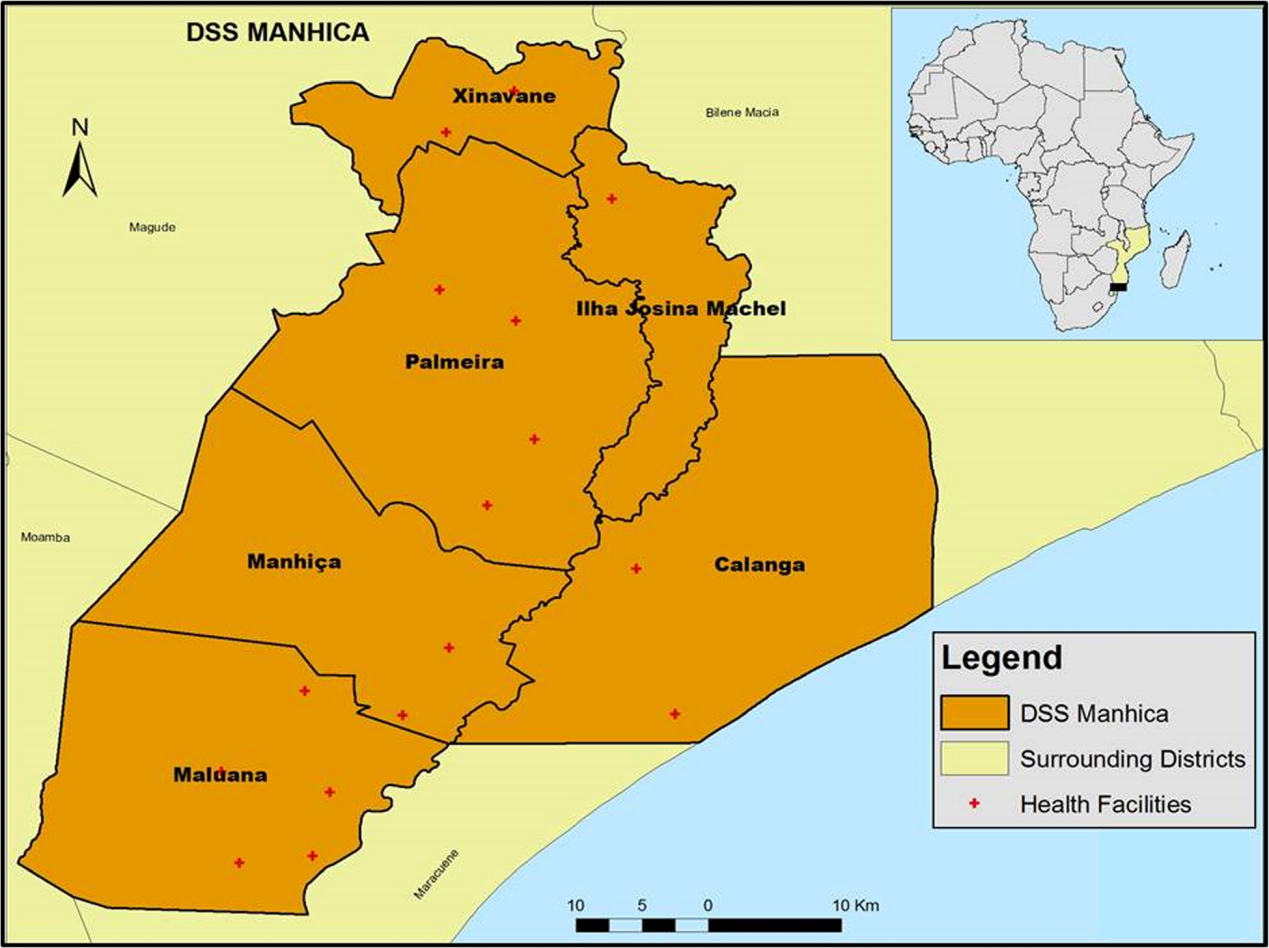
Administrative Division of the district of Manhiça (Source: HDSS-CISM). Demography Department, Manhiça Health Research Centre. Map created using ArcGIS

In Mozambique, the study was conducted in the district of Manhiça (Fig. 1), a semi-rural district covering an area of 2360 Km^2^ and located ~90Km from Maputo, the capital city of Mozambique. The study was implemented by the Manhiça Health Research Centre (CISM), which runs a Health and Demographic Surveillance System (HDSS) covering a population of 198,000 inhabitants registered as permanent residents and distributed across 44,000 households. Within the HDSS study area there are 12 health centres and two hospitals; the Manhiça District Hospital (MDH) and Xinavane Rural Hospital (see Fig. 1) [37]_._

### Study population

The study population are community members that live in the district of Manhiça. The main town (Manhiça Sede) has been a municipality since 1998. The majority of the population lives in rural areas and most are engaged in subsistence farming, labour work in sugar cane plantations and sugar refining companies, and small-scale businesses. The population is mainly from the Xironga and Xichangana ethnic groups. Nearly half of all women (47%) and a quarter of all men (24%) are illiterate [38]. The study was conducted in four administrative posts (Manhiça sede, 3 de Fevereiro, Xinavane and Maluana) to enhance geographical variety. Community members were randomly selected within the HDSS area based on specific criteria for each activity.

### Data collection

In Mozambique, four focus group discussions (FGDs) and 16 In-depth interviews (IDIs) were conducted between April and May 2017. The use of IDIs and FGDs was purposively chosen for this analysis in order to gain an in-depth understanding of the topic through triangulation. The IDIs and FGDs were conducted by two trained social science research assistants with a combination of medical and social science backgrounds to ensure a good balance between the capacity to engage in in-depth discussions on social behavioural aspects and being familiar with the biomedical implications of the topics of interest.

#### In-depth interviews

This technique allowed the interviewers to maintain the privacy and confidentially of the participants and allowed them to comfortably express their opinions, knowledges, experiances, and practices regarding antibiotics in detail without the pressure of a group [39]*.* These interviews were conducted one-on-one with community members that included mothers of children under 5 years old as well other adult male and female participants (aged > 18) regardless of parenthood status (see Table 1). The IDIs were performed at a time and location that provided sufficient privacy; they lasted about 15–30 min. They consisted of open-ended questions that explored the following themes: *knowledge and awareness of antibiotics, practices of antibiotic use, knowledge and awareness of antibiotic resistance.* Three close-ended questions were used in addition as tools to test the consistency of individual participants’ knowledge regarding the definition and purpose of antibiotics. Further, the interviewer displayed a set of show-cards with the aim of assessing participants’ capacity to visually recognize antibiotics among three commonly available pills in the study area: an analgesic ‘Paracetamol’- (circular white tablet with slot), a non-steroidal anti-inflammatory drug ‘Ibuprofen’ (circular red tablet without slot), and an antibiotic ‘Amoxicillin’ (capsule with red and yellow lids. In addition, the interviewer displayed show-cards to assess recognition of any antibiotic among five different antibiotics that are commonly available in Manhiça: Amoxicillin (capsule with red and yellow lids and a suspension bottle), Metronidazole (circular yellow tablet with slot), Co-trimoxazole (circular white tablet with slot), Erythromycin (capsule with red and yellow lids) and Azithromycin (capsules with white lids). If antibiotics were recognized, participants were asked whether they or their relatives had ever obtained the antibiotics and the conditions they aimed to treat.

**Table 1:** Target population - In-depth interviews

Target population	Number of participants
Mothers of children under 5 years old	8
Males ≥18 and < 60 years old	2
Females ≥18 and < 60 years old	2
Males ≥60 years old	2
Females ≥60 years old	2
Total	16

#### Focus group discussions

The focus group discussions generated discussions and debates about the topic discussed at hand from participant’s own views [40]. Each discussion involved 6–8 participants with similar characteristics including males and females from > 18 to < 30 and > 30 years old (see Table 2). The FGDs were performed by two research assistants, one moderator and one observer who took notes of group dynamics and non-verbal communication. The FGD took place in a location previously agreed with the participants and lasted approximately 40 min. The FGD guide was structured around four thematic sections, namely: *access to treatment*; *suppliers of medicines*; *knowledge and practices regarding antibiotics; and antibiotic resistance.* The first two sections consisted of open-ended questions. The third section started with an open-ended question about knowledge of antibiotics supported by the use of show-cards to assess the recognition of five antibiotics (the same exercise conducted in the IDI). For the fourth section, the guide contained a hypothetical case scenario illustrating a concrete case of resistance upon which participants were encouraged to react, comment and discuss. Finally, participants were asked if they had ever heard of antibiotic resistance, its causes and implications.

**Table 2:** Target population - Focus group discussions

Target population	Geographical area within the HDSS	Number of participants
Females ≥18 and < 30 years old	Xinavane	6
Males ≥18 and < 30 years old	Maluana	6
Females ≥30 years old	Manhiça Sede	6
Males ≥30 years old	3 de Fevereiro	8
Total		26

### Recruitment of study participants

Different groups were selected through stratified random sampling covering the 4 administrative posts. The IDIs participants consisted of 8 mothers of children under 5 years of age, and a group of non-parents: two females and two males ≥18 and < 60 years old and two females and two males ≥60 years old. For the selection of this target group, stratified random sampling was applied whereby two participants that met the criteria were randomly selected from each Administrative Post based on the HDSS database. The FGDs participants were also selected through stratified random sampling, however this time sampling was based on age and gender and did not require a proportional distribution amongst Administrative Posts.

The FGD sampling frame encompassed all individuals registered in the HDSS with study specific inclusion criteria. The study intended to convey four distinct groups of participants, namely: Females ≥18 and < 30 years old, Males ≥18 and < 30 years old, Females ≥30 years old and Males ≥30 years old. Each target group was randomly matched to one of the four Administrative Posts (see Table 2), given that the number of target groups matched the number of existing Administrative posts [4]. A list of potential participants that matched the target group criteria was generated from the HDSS of each of the selected Administrative Posts. The required number of FGD discussants was randomly selected based on the lists, including an excess of 20% to account for refusals, deaths, migrations and exclusion criteria.

The research assistants approached the selected participants for the IDIs and FGDs after identifying their HDSS house number with the help of the local community leader. The participants were invited to participate in the IDI or FGD after being informed of the purpose of the study, and the anonymous character of the activities. For the FGDs the time and place were suggested by the research assistant and agreed with the participants.

It was required that all FGD participants came from different households to minimize clustering of ideas and opinions. Community members who participated in IDIs were not eligible for inclusion in FGDs and vice-versa. After conveying the group, eligible participants were once again informed of the purpose of the FGD, this time through a written participant information sheet attached to an informed consent form that was handed to the participants. Simultaneously, the contents of the informed consent were read out loud to the participants who were invited to ask questions in order to ascertain comprehension prior to requesting them to sign it.

### Data management and analysis

In-depth interviews and FGDs were conducted either in Changana (the local dialect) or in Portuguese (the official language) depending on participants’ preference and were audio-recorded using digital voice recorders. Recorded data was archived in digital format and was transcribed *verbatim* and simultaneously translated to Portuguese, when necessary, by two trained social science research assistants who conducted the interviews. In order to ensure consistency and accuracy, the most experienced team member (senior encoder) carried out quality control by checking the audio with the transcription to identify and then amend any error that emerged. In addition, a matrix was designed, where the rows represented the reference of the participant and the columns the questions addressed (organized into pre-determined and emerging themes. Quotes from participants’ answers where inserted in the corresponding cells. The qualitative data was analysed manually following a combination of content and thematic analysis. The content analysis [41] began by organising and categorising the data based on pre-determined themes (the same ones that guided the data collection process), such as ‘awareness of antibiotics’, ‘visual recognition of antibiotics’ or ‘knowledge of antibiotic resistance’. Within these themes some subcategories were generated (thematic analysis), such as ‘discarding or returning medication’, ‘storing medicines for future use’ and ‘sharing medicines’, all under ‘practices’, which were directly linked to knowledge and practices. The process of data immersion led to the identification of emergent themes (thematic analysis) [42], particularly regarding local understandings of antibiotic resistance. All themes were contrasted with the data to test their consistency as well as to identify points of tension and divergence among the different data collection sources and participants (triangulation) [43]. The presentation of the qualitative data (interviews and FGDs) complied with the SRQR guidelines for reporting qualitative research [44].

### Ethical considerations

All data collection was conducted after obtaining voluntary, signed informed consent from each participant, as well as permission to record individual interviews and group discussions. Ethical approval for the study was obtained from CISM’s Institutional Review Board (approval ref.: CISM/466/0316)**,** followed by administrative approval by the Mozambican Ministry of Health (approval ref.: 1487/GMS/002/016). All participants were de-identified by only linking them to their DSS unique identification numbers to guarantee confidentiality.

## Results

The results are presented in descriptive format under four major themes extracted from the IDIs and FGDs: (i) General knowledge of antibiotics; (ii) Visual recognition of antibiotics; (iii) Antibiotic use; (iv) Awareness of antibiotic resistance. Quotes from the respondents within each category are presented to illustrate the findings.

### Participants’ characteristics

A total of 16 community members were recruited for IDIs, 12 of the participants were female. The age of participants were between 18 and 29 (*n* = 5), between 30 and 59 (*n* = 6) and over 60 years (n = 5). With regards to education, 5 participants had received no education, and the remaining 9 participants did not completed secondary school and 2 did not respond. Of these respondents 9 were literate and 5 illiterate. Domestic workers and farmers made up the majority of participants occupations (n = 6 and *n* = 7 respectively) (see Table 3).

**Table 3:** Background characteristics of the 16 community members

	Frequency n (%)	
	n	(%)
Sex		
Male	4	25.0
Female	12	75.0
Subtotal	16	100
Age range (years)		
(18 to 35)	11	68.8
(36 to78)	5	31.3
Subtotal	16	100
Village of residence		
Xinavane	4	25.0
3 de Fevereiro	6	37.5
Manhiça sede	6	37.5
Subtotal	16	100
Level of education ^a^		
None	5	35.7
Incomplete primary school	3	21.4
Primary school	3	21.4
Subtotal	14	100
Able to read?		
Yes	9	56.3
No	7	43.8
Subtotal	16	100
Occupation ^b^		
Domestic	6	40.0
Commerce	1	6.7
Farmer	7	46.6
Subtotal	15	100

^a^ Two participants did not provide the level of education

^b^ One participant did not provide the occupation

A total of 26 participants were recruited for the FGDs, with 6–8 participants in each of the four groups. The groups were 1) adult females younger than 30, 2) adult females older than 30, 3) adult males younger than 30 and 4) adult males older than 30 (see Table 4).

**Table 4:** Background characteristics of the community members focus group discussion

	Number	Percent
Sex		
Male	12	47
Female	14	53
Subtotal	26	100
Age range		
Young (18 < 30)	12	47
Adults (> 30)	14	53
Subtotal	26	100
Place of residence		
Xinavane	6	23
3 de Fevereiro	8	31
Manhiça sede	6	23
Maluana	6	23
Subtotal	26	100

### General knowledge of antibiotics

The questions on awareness and knowledge regarding antibiotics begun by examining how participants freely conceptualised “antibiotics”, including their recollection of the term antibiotic, as well as their version of the definition, the attributes and utility of the drug. The data revealed mixed responses regarding familiarization with antibiotics. The majority of participants who claimed to be familiar with antibiotics were able to provide a description of this class of drugs. The group of men ***≥*** 30 years old gave the most detailed description of antibiotics, describing antibiotics visually as pills in the form of capsules with red and yellow lids. Of note, participants in this group only conceived of antibiotics as tablets or capsules, disregarding the possibility of other presentations, such as oral suspension, injection or cream.

*P3: I also know them...*

*P3: They are capsules*

*P2: I know antibiotics [cacophony]*

*P8: Me too*

*I: Are you able to describe what antibiotics are?*

*P8: Antibiotics are ‘Amoxilina’ [Amoxicillin]*

*P3: But we do not know them by name. They give us those, if they are [those] or not, we have no way of knowing...*

*P3: They have two colours (FGD,****≥****30 years old participants)*

When questioned about the usage of antibiotics, the majority of the participants that had heard of the term were able to indicate the conditions they perceived to be treated by antibiotics, which were: malaria, infections, sexually transmitted diseases, tuberculosis, cough, fever, body pain, stomach pain, wounds and pain in the uterus, wounds, toothache, headache, illnesses related to the bones, rheumatism, dermatological problems, foot ailments, constipation, dysentery, and mouth defects. The vast majority of the participants in this study cited more than one condition perceived to be treatable by antibiotics.

*Antibiotics, well I have only heard the name. They are normally for pains like, STDs, wounds … you see? Things like that is my understanding. (IDI,* ≥*30 years old participant)*

The remaining participants that had also heard of antibiotics were not able to describe them in any sense and claimed they did not know what they were for. Although the majority of participants understood antibiotics as medicines, it should be pointed out that one participant understood antibiotics as a disease and claimed to have never heard of antibiotics because he never got ill.

*I am not really sure, I only know there are pills that tackle infections, but knowing that this is an antibiotic, that that is an antibiotic … I do not know the qualities of the pills. (IDI,* ≥30 years old *participant)*

The perceived definition and purpose of antibiotics was further captured through three close-ended questions. When asked what antibiotics were, with the intention to lead participants to talk about their purpose, there were mixed responses, with some of the respondents reporting that they did not know what antibiotics were, while a few participants responded that antibiotics were drugs against infection and others thought that they were drugs that served to combat high blood pressure. Furthermore, the majority of respondents reported that they did not know what antibiotics do, while only a small minority stated that they kill bacteria. In response to a question surrounding antibiotic usage, most of the respondents said they did not know what antibiotics should be used for, whilst few answered that they should be taken to treat bladder infections, muscle pain or weakness.

It is important to note that none of the respondents of the three closed-ended questions provided consistently -sound answers, meaning that even those that did provide a correct answer, then gave a contradicting answer to the following question. For example, of the two participants that correctly indicated that antibiotics are used to kill bacteria, both went on to give wrong answers to other questions related to knowledge and purpose of antibiotics. These responses therefore indicate inconsistency in participants’ understanding of antibiotics, with each seeming to answer each question arbitrarily (see Table 5).

**Table 5:** Multiple-choice questions results

Questions	Optional answers	Response frequency	Percent
What do you think are antibiotics?	Painkiller	1	6%
	Drug against fatigue	1	6%
	Drug against high blood pressure	3	19%
	Drug against infection	3	19%
	Don’t know	8	50%
	**Subtotal**	16	100%
What do you think antibiotics do?	Decrease blood pressure	0	0%
	Give energy	1	6%
	Kill bacteria	2	13%
	Stop pain	4	25%
	Don’t know	9	56%
	**Subtotal**	16	100%
When do you think antibiotics should be taken?	Bladder infection	3	19%
	Muscle pain	2	13%
	Weakness	1	6%
	Headache	0	0%
	Don’t know	10	62%
	**Subtotal**	16	100%

### Visual recognition of antibiotics

In order to assess the extent to which participants meant what they were verbalizing when referring to antibiotics, two exercises using show-cards were displayed. In the first show card, participants were challenged to visually identify an antibiotic among three commonly available pills. A considerable number correctly identified the picture of the antibiotic (in this case, Amoxicillin), and others pointed to the picture of Paracetamol (see Table 6).

**Table 6:** Responses to show-cards

Questions	Optional answers	Response frequency	Percent
First show-card: Indicate which of the pills is the AB	Photo of Paracetamol	5	31%
	Photo of non-steroid anti-inflammatory drug	1	6%
	Photo of antibiotic	7	44%
	Don’t know	3	19%
	**Subtotal**	16	100%
Second show-card: Does the participant recognize any of the antibiotics?	Yes	12	75%
	No	4	25%
	**Subtotal**	16	100%
*If yes,* has the participant ever obtained antibiotics?	Yes	12	75%
	No	4	25%
	**Subtotal**	16	100%

It is important to note that the 75% of respondents that had previously mentioned to have already heard of antibiotics correctly selected the photo of Amoxicillin, supporting the presumption that amongst those who claimed to know about antibiotics the mixing-up of this concept with another class of drugs was less likely. These participants consistently claimed to be able to identify the antibiotic based on the shape (capsules) and colours (red and yellow). Among those who initially responded that they had not heard of antibiotics, the majority did not visually identify antibiotics, however a few correctly did identify them.

When the second show-card with photos of five different antibiotics was displayed (Amoxicillin, Metronidazole, Co-trimoxazole, Erythromycin and Azithromycin), none of the participants recognised all five as antibiotics. The vast majority visually recognized 1–3 of the antibiotics, with Amoxicillin being repeatedly the most frequently recognised. To most of them, the basis for the recognition of any antibiotic was their own, or their relatives’, experience of acquiring and using antibiotics (see Table 6).

### Antibiotic use

Both the FDGs and the IDIs dedicated time to discuss practices around antibiotic use but although questions and probes referred to antibiotics specifically, participants mostly spoke about ‘medicines’ or ‘pills’ in general. This aspect, coupled with the insights drawn from the previous exercises, made clear that when discussing practices around antibiotic usage participants were inasmuch referring to medicines in general as to antibiotics in particular. Five themes pertaining to common antibiotic use practices were identified as follows.

#### Reasons for not purchasing the full course of medication

Participants agreed that not purchasing the full course of medication was a common practice. According to them, unavailability of the given medicines at the health facility (where medicines are subsidized), led patients to resort to private pharmacies, which are less affordable. As a result, patients did not purchase the full course. Therefore, medicines being out of stock and lack of money were reported as factors resulting in purchasing an incomplete course of treatment.

#### Self-medication

Some participants acknowledged that self-medication is a common practice, particularly with over-the-counter medication, such as ‘Paracetamol’. They explained that sometimes one might simply have a headache which, in their view, does not require going to the hospital. Participants also mentioned cold or flu and stomach-ache as conditions not requiring hospital assistance and suitable for home treatment. However they did not specify the type of medication used in this instance therefore antibiotics could be one of the choices since participants used the terms antibiotic and Paracetamol interchangeably when asked about their self-medicating practices regarding antibiotics. Again, this suggests that the term ‘antibiotic’ may have been used among participants to describe drugs in general, rather than just antibiotic drugs.

#### Adherence to treatment

When asked about in-depth adherence, participants recognized the importance of taking the full course of medications and finalizing treatment as instructed. Few respondents reported quitting the medication before the prescribed treatment was completed. However, they claimed it was important that the patient felt good before they did so. Only one participant stated they stopped due to the “bitterness” of the drug. Participants generally perceive that the health care provider plays an important role in advising on treatment compliance.

*I won’t deny it, sometimes I also do that [laughter], I take pills and when I see I am better, especially if the pills are bitter, I stop taking them, I quit. (FGD, 18 – 29 years old participants)*

#### Storing medicines for future use

Participants unanimously denied that they buy a larger amount of medicines than those prescribed (including, but not exclusively, antibiotics). However, the majority of participants eventually disclosed that they do recurrently store medicines for future use. According to them, there are some medicines that health providers often recommend, which can be dispensed in excess because the pre-packaged amount of drugs within one unit (e.g. a blister pack of tablets) often exceeds the maximum amount prescribed. In those instances, participants referred that they keep them in case themselves or relatives present the same symptoms or disease. Leftover medicines are perceived to be mostly due to interrupted medication rather than purchasing drugs in excess.

*Yes, I buy it to take it until it finishes [but] when there is some left and I already feel good, I keep it to take [it] again. (IDI, ≥18 participant)*

It was noticed that those participants who do keep medicines for future use claim to only take them if it is within the expiration date. The expiration date was mentioned as important criteria to consider when taking medicines stored at home. Interestingly, most of the respondents reported that it is common practice to first take the medicines at hand and only after seek attention at the hospital.

*I keep them so that if I become ill again, if I have them I take them … if when I take them they do not have the same effect, [if] I feel I do not get better even taking them [stored medication] then I go to the hospital. (IDI, ≥18 participant)*

#### Sharing medicines

Despite the suggestion that in this setting there is a practice of storing medicines for future use, most participants claimed that they do not share with, nor receive medicines from, others, mainly because they cannot tell whether the disease or condition to be treated is the same. The need to go to the hospital for adequate diagnosis and receive the appropriate medicines was repeatedly emphasized.

Yet some participants did disclose that sharing medication is a common practice and that they are used to share with, and receive medicines from, others, in particular relatives and other household members.

*Here at home we are all siblings, we all have children [so] when they have [medication] or I also have and my son also starts with it [symptoms], they give me and I make him take it. (IDI, ≥18 participant)*

They identified two cases in which sharing medication is justified: compatible symptoms and conditions perceived as non-threatening. Regarding compatible symptoms, this referred to cases in which medication was shared because the receiving person had symptoms similar to those for which the medication had originally been prescribed and used. On the other hand, participants argued that some non-threatening conditions, such as headaches, could be treated with stored medication that did not require prescription.

#### Discarding or returning medication

Participants who claimed not to store medicines for future use revealed the habit of throwing away the remaining medicines or, in a few cases, of returning them to the hospital. One important factor mentioned was that if the medicine expired it could cause other diseases or even death, besides being potentially dangerous for children’s health in the event of unsupervised consumption.

*When there are left-overs [medication], those from the hospital, we say those are no longer useful so we throw them away because then when you go take them they can make you ill, even die, because it is rotten. (FGD,* ≥*30 years old participants)*

### Awareness of antibiotic resistance

When asked directly if they had heard of antibiotic resistance, respondents consistently stated that they had never heard of the term. Amongst the few that did recognize it, it was argued that antibiotic resistance happens when either the disease or the body resists the effects of the medication.

The hypothetical case scenario featuring a patient seeking medical care at a hospital helped probe further participants’ perceptions of the phenomenon. When presented with this scenario, the majority of participants were familiar with the illustrated phenomenon, even if they could not attribute a specific name to it.

Hypothetical case scenario:

“A person living in this community had a certain health condition. He went to the hospital and was given some the most appropriate medication for his condition. He took the full course of the medicine but he did not feel better. A few days later he went to another hospital for the same condition and he was given the same medicine that he was given at the first hospital. Once again, he took the full course of the medicine as the doctor instructed and still did not feel better.”

Overall, they discussed a variety of factors that could be causing the treatment to fail repeatedly, which branched into four overarching categories: 1) behavioural, 2) physiological, 3) pharmacological and 4) pathological.

#### Behavioural: resistance as a result of sub-optimal adherence

Participants were aware of the risk of treatment failure and they understood that one of the main factors behind this was sub-optimal adherence. They expressed doubts about the extent to which what other people report in terms of adherence correlates with what they do in practice, and felt that it is a common practice not to adhere with the medical prescription provided, even if a patient claims to do so. Therefore, even though the presented scenario described a case in which adherence was practiced, most of the discussions revolved around the failure to do so as a crucial factor leading to treatment failure. Sub-optimal adherence was understood as not observing the prescribed duration of the treatment, and not adhering to requirements such as avoiding alcoholic drinks and respecting the validity date.

Along the same lines, in the context of treatment success, participants recognised the importance of observing the treatment timeframe and mentioned “interruption of treatment”, specifically as soon as symptoms decrease or disappear, as a behaviour that can lead to treatment failure. Taking medications on an empty stomach was also mentioned as a factor compromising treatment efficacy, manifested by the patient not getting better or being subject to additional health problems, even if the medicine is taken as prescribed. According to one participant, *“this disease will stay with the person forever, even if she takes medication correctly”*. *(FGD, ≥ 30 years old participants).*

When asked about the most appropriate conduct, respondents recognised the hospital as the source of correct treatment and emphasized the need to comply with the treatment in order to avoid treatment failure.

*I would seek the prescription first at the doctor [isn’t it?]... An authorized person, so they could dictate [give orientation] it to me properly because if I go to buy I will not know how to use ... I will use it recklessly I will create more problems than those [laughs] I had. (FGD, ≥ 60 years participants)*

#### Physiological: resistance originating in the person’s body

Despite the fact that behavioural factors were the most cited, one group (females ≥18 and < 30 years old) considered that the problem illustrated in the hypothetical scenario was not behavioural but rather related to the individual’s body. In addition, participants understand that each body reacts differently, that there are particular individual characteristics (allergies, intolerances, simultaneous presence of distinct diseases) that lead to resistance.

*But this is not the issue, that the pills are of poor quality, that they don’t work, no, they do work but it depends on each person’s organism. (FGD, 18–29 years old participants)*

#### Pharmacological: resistance as a result of ineffective medication

After discussing the hypothetical scenario, a few participants in the male grou*p (*≥*30 years)*, perceived the cause of the resistance to treatment to be rooted in the medication itself. These participants considered that, in some cases, the medications they are given are ineffective and are the origin of treatment failure. In this sense, the treatment itself is considered the source of its failure.

*We had those pills they called Rosoquina, Rosoquininas, that did not do us good because when one took them, often, it would cause an itch and it would seem like one was cured even though they had not worked. (FGD, ≥30 years participants)*

#### Pathological: disease as the source of resistance

One participant referred to resistance as a phenomenon that takes place in the disease itself, as a property of certain diseases that are resistant to treatment and thus lead to its failure:

*This is what causes the person to die, take the medicines, pick up those diseases that are resisting, they don’t go away, at last [you] die, [while] taking those antibiotic tablets, the ones they say that they are Amoxilina (Amoxicillin). (FGD, ≥30 years participants)*

This was the only participant that specifically referred to antibiotics, as well as providing the name of a concrete antibiotic (Amoxicillin), when discussing resistance to treatment success.

## Discussion

To the best of our knowledge, this is the first qualitative study undertaken in Mozambique aiming to describe the different dimensions of community knowledge of antibiotics and antibiotic resistance, as well as practices regarding antibiotic use. This aligns with a recent statement regarding the scarcity of evidence on self-medication and other aspects of antibiotic use in Africa [45].

In this study, we identified key dimensions that situate participants’ knowledge of antibiotics (or lack thereof): i) those who visually recognized antibiotics, were aware of the term “antibiotic” and could provide some definition based on the colour, shape and the illnesses they perceived were treatable through antibiotics (even if inaccurate), ii) those who visually recognized antibiotics but were unaware of the term nor could they provide any definition linked to it, iii) those who could not visually recognize it, nor were familiar with the term, nor could define it in any way. The majority of participants fell into the first group.

The results from the different attempts to support participants in conceptualizing antibiotics in their own terms suggest that there is limited knowledge of the concept “antibiotic”, as none of the participants provided an elaborate and consistent definition of antibiotics that does not contradict biomedical knowledge. Their conceptualisation seems to have been limited by the characterisation of the medicine based on the appearance and use of the dominant antibiotic class in this setting *(Amoxicillin*) which is provided as a distinct red-yellow capsule and is also named ‘red & yellow’. Poor knowledge of antibiotics among the general population has been reported in different studies in LMICs [23, 46, 47] as well as in and high-income countries (HICs) and amongst European populations [18]. Yet in contrast to our study, these studies assessing the knowledge of antibiotics focused on their use and purpose. Our study went further by examining participants’ familiarization with the term and assessing the extent to which they made a connection between the term and a definition, be it in terms of common characteristics, use and purpose, or other features. This effort also ensured, as much as possible, that both interviewers and respondents meant the same thing when they were referring to antibiotics.

The limited understanding of the treatment indications of antibiotics found in this study was consistent with existing literature [6, 20, 47, 48]. This was evidenced by the fact that the vast majority were unable to specify the type of agent that antibiotics are effective against, and also that the range of diseases mentioned to be treatable by antibiotics was beyond the scope of antibiotic indication. This finding correlates with studies conducted mostly in high and middle income countries (including in Indonesia, Albania, Iran and the UK) that evaluated lay knowledge regarding antibiotic use [15, 47, 49, 50]. Considering the literacy limitations and low exposure to information, amongst other factors, that act as barriers to health literacy in poor-resourced countries such as Mozambique, our results regarding the limited knowledge of antibiotics are not surprising [3, 17, 46]. Moreover, although this community has a relatively high coverage of health services compared to other regions of the country, it is not likely that such knowledge would be gained through their interaction with the healthcare providers. Providers in such settings are not instructed nor prepared to engage in dialogues with patients beyond naming the drugs and the conditions they are to treat. It is unlikely that providers will explain biomedical classifications be it in terms of pathogens (i.e., ‘bacteria’) or descriptions of classes of drugs (i.e. ‘antibiotics’). This limited exchange has also been found in other settings. A cross sectional study in Malaysia shown that other general terms (such as germs) are normally used during counselling, instead of the microbiological term “bacteria” [19].

Despite the fact that antibiotics in Mozambique may be presented in different pharmaceutical forms or capsules of colours which may confuse the users, one important finding was the ability of some participants to define antibiotics, influenced by their familiarisation with Amoxicillin, which locally is typically presented in the form of yellow and red tipped capsules. This finding is concordant to the recent research conducted by Torres (2019) in Mozambique, which reported that participants reported to know Amoxicillin, describing the capsules with in red and yellow colour [30]. Apart from limiting their conceptualisation to an attempt to characterise Amoxicillin, they were not able to conceptually distinguish antibiotics from other types of medicines. The link to Amoxicillin may be attributed to the ample availability and use of this antibiotic, as reported from studies in different African countries as well as Mozambique [1, 11, 21, 27, 51–56]. This finding illustrates that if antibiotics have a distinct appearance, this may aid in recognition and improve use.

Looking at perceptions of the treatment purpose of antibiotics, participants mentioned a variety of conditions, including those that are not treatable by antibiotics. Subtle misconceptions like this could lead to inappropriate use and potentially precipitate and/or sustain the problem of resistance [3, 19, 20]. The results are comparable to studies conducted in other LMICs [45] and HICs [18], but also add conditions that were not found in other studies, such as: malaria, pain in the uterus, and mouth defects.

In alignment to studies in other settings [6, 32, 47, 57], self-medication in addition to sharing of medication, particularly between family members, was reported to be a common practice in the studied communities, especially if the condition was perceived as non-threatening, and, in the case of sharing, if symptoms were perceived as compatible among those sharing the drug. It is likely that such practices, reported for medicines in general, are also applicable to antibiotics considering that the term antibiotic was used interchangeably with the term ‘medicines’ when discussing these practices.

None of the participants in the individual interviews seemed to be familiar with the term or the concept of antibiotic resistance, yet the dynamics during FGDs (which relied on participants reacting to the presentation of a “resistance” case scenario), allowed participants to relate to the phenomenon and elaborate on it, even though practically no one had heard the term before. This is in accordance with a study conducted in India, which found that none of the participants recognised the term “antibiotic resistance”, but following a brief description, some participants were familiar with the concept [46]. Respondents in our study understood the phenomenon at a practical level (in terms of how it manifests and plays out) and blamed resistance on several forms of patient misbehaviour, generally classified as sub-optimal adherence to medical instructions, but also on circumstantial aspects such as taking medication on an empty stomach, which is not aligned with the biomedical concept of resistance. On the other hand, some participants related resistance to the treatment itself not being effective due to physiological limitations of the body (i.e., its inability to respond to the drug), ineffectiveness of the medication itself or resistance of the diseases, regardless of individual behaviour.

The various discussions with participants consistently revealed that they do not associate the pathogenic agent of the disease into the equation of antibiotic resistance, as, in their view, it is either the result of misuse, or it is the body, the disease or the drug itself that becomes resistant, leading to treatment failure. Among these conceptions, some alignment with biomedicine was revealed with regards to the concept of antibiotic stewardship, in which misuse leads to resistance. However, the majority of respondents only seen at an individual level and not at a macro, environmental level. In other words, antibiotic resistance affecting an individual is blamed on that individual’s inappropriate behaviour and framed as a phenomenon whose cause and effects are located exclusively within that individual. In this way, other factors that could lead to resistance are ruled out, including the possibility of being infected by already carrying resistance to a specific antibiotic. Attribution of antibiotic resistance to the individual level, rather than an acquired property of bacteria was also found in a study conducted in 9 European cities [58].

Regarding stewardship, the finding on the gap between participants’ theoretical knowledge of appropriate antibiotic use and their behaviour is consistent with studies conducted in high-income countries, such as a large household survey in Great Britain that found that 87% of respondents who did not complete their last course also said that a course of antibiotic should always be completed [15].

There is extensive lay knowledge built around exposure to treatments and medicines, mostly as a result of people’s own experience and the interpretation given to such experience; this non-medical knowledge shows that lay people do think about and conceptualize drugs in general, within which they fit concepts around antibiotics and resistance in terms that are intelligible to their own realities. In order to better reach communities and improve community awareness it is important to reflect on how lay people conceptualize resistance, considering that they do not yet differentiate antibiotics from other classes of medicines, nor do they understand fully the indications of this class of drugs, even if to some extent they are able to visually recognize a common antibiotic by relating its image to the term ‘antibiotic’. Future campaigns need to consider these lay understandings and tackle gaps in knowledge or misinformation. This disparity between lay and biomedical concepts is not to say that a higher level of knowledge is not possible to attain in our settings. In fact, the HIV/AIDS epidemic has proven that complex terms such as “virus” and “antiretroviral” were certainly discussed with literacy-limited community members [59–62]. A parallel could be made with regards to the incorporation of terminologies related to “bacteria” and “antibiotics” in efforts to improve knowledge and practices to combat antibiotic resistance.

Lastly, health policies should also consider the structural problems pointed out in this study, including self-medication which is a result of easy accessibility, and practices around sharing medications which is potentially a result of self-diagnosis and misinformation at the level of the supplier, be this at the hospital or pharmacy. Given the demand side issues highlighted in this study it is crucial for health providers to take more responsibility in controlling access to antibiotics. Nevertheless, the existing trust that participants in this study expressed with regards to formal antibiotic providers represents an opportunity for developing and targeting specific educational interventions and to inform policy to promote the appropriate use of antibiotics.

### Study limitations

Being a qualitative study, the strength of the study was the level of depth and multiple dimensions captured with regards to knowledge of antibiotics and antibiotic resistance. Nonetheless, the study was designed to capture people’s ranges in perceptions and not the magnitude of the problem. Therefore, it has not captured how extensive or recurrent these constructs and practices are but rather what people consider to be common. As we pointed out, we cannot guarantee that participants were always referring to antibiotics throughout the interviews and discussions since, in spite of the regular use of prompts and visual aids to help focus the conversation on antibiotics, respondents would often interchangeably make reference to antibiotics and other medicines, conflating the two. Thus, the depth of some discussions where the term “antibiotics” was used were limited due the fact that participants did not seem to express confidence in their reference to antibiotic. Additionally, the fact that participants knew the study was related to antibiotics may have induced them to use the term “antibiotic” more than they would have in normal conversation when talking about medications. An additional limitation of this study is the fact that, despite the effort to discuss “antibiotic resistance” by making use of a hypothetical scenario, participants did not use the term “resistance to antibiotics”.

## Conclusion

Limited and inconsistent knowledge about antibiotics and antibiotic resistance were the main findings of this study. However, participants did build some constructs around these concepts, namely that antibiotics (mainly Amoxicillin) are yellow and red capsules used to treat a non-specific range of complaints, and that resistance refers to the effects of sub-optimal adherence and behavioural, physiological, pharmacological and pathological factors. These constructs were built on the basis of their lived experiences of antibiotic usage. Treatment failure is conceptualised at the individual level, not as a public health concern. In addition, in spite of awareness of the importance of medication compliance, participants acknowledge it is common to not abide by it. Thus, further research to understand this discrepancy between the knowledge of responsible medication compliance and current practices, as well as tailored interventions that tackle inappropriate use and behavioural change, are crucial and should be prioritized. The existing trust in drug providers represents an opportunity to train and use drug providers as a medium to communicate accurate information about antibiotic use and resistance.

## Supplementary information

**Additional file 1.**

**Additional file 2.**

**Additional file 3.**

**Additional file 4.**

## Data Availability

All the data and materials used to analyse and interpret the results of the study are available from the country study investigators on reasonable request.

## Abbreviations

ABACUS: Antibiotic access and use study
AMR: Antimicrobial resistance
CISM: Manhiça Health Research Centre
FGD: Focus group discussion
HDSS: Demographic surveillance system
HICs: High-income countries
IDI: In-depth interview
IEC: Information, education and communication
KAP: Knowledge, attitudes and practice
LMICs: Low and middle-income countries
MDH: Manhiça District Hospital
WHO: World Health Organization
