# Supplementary material for: Community knowledge and practices regarding antibiotic use in rural Mozambique: where is the starting point for prevention of antibiotic resistance?
Source: BMC Public Health. 2020 Jul 29;20:1183. doi: 10.1186/s12889-020-09243-x (PMC7389384; doi:10.1186/s12889-020-09243-x)
Supplement: Supplementary file 2 — Additional file 2. [file 12889_2020_9243_MOESM2_ESM.pdf]

cism centro de investigação em saúde de manhiça 20 anos	**Appendix P. Preparatory_community member_FGD guide** "Antibiotic access and use in low- and middle-income countries" Acronym: ABACUS.	INDEPTH Network Better Health Information for Better Health Policy

Instructions for the interviewer

Per study site, for each of the 6 preparatory FGDs, 6 to 8 community members will be recruited based on the HDSS database. Participants of a certain focus group may know each other, but should all come from different households. Because the preparatory FGDs should represent community norms, antibiotic suppliers and healthcare workers will be excluded from participation.

Stratification focus groups

Focus group category	Geographical area within the HDSS	Preparatory FGDs in phase 2 (number of participants)
Females ≥18 and <30 years old	A	1 (6-8)
Males ≥18 and <30 years old	B	1 (6-8)
Females ≥ 30 years old	C	1 (6-8)
Males ≥ 30 years old	D	1 (6-8)
Adult group specified by local PI according to local context	Unspecified	1 (6-8)
Adult group specified by local PI according to local context	Unspecified	1 (6-8)
Total		6 (36-48)

FGD: focus group discussion.

The first 4 preparatory focus groups are stratified by gender and age according to the table below, and will take place in 4 different geographical areas in the HDSS. As such, individuals who participate in a certain FGD should all live in the same geographical area where the FGD is held. Inclusion of participants in the other 2 focus groups will be based on social groupings relevant to the local setting, as specified by the local PI.

The focus group discussions will be performed by 2 HDSS research assistants. One moderator will lead the discussions, while another person will be taking notes. We aim to capture all the different views that are present among the members in a particular focus group.

Informant data:

NOTE TO MODERATOR: Before starting the FGD, collect details for each participant (age, sex, and occupation of all participants; location of FGD) in the separate eCRF.

Accessing treatment

1) Please describe the different alternatives that people in this community use to receive treatment if they are ill (i.e. health centre, pharmacy, traditional healer, medicine peddler, etc). What are the main challenges people face in accessing these facilities in terms of mode of transport, time, and cost?
2) In general, how would you describe the *quality* of the health services (public and private, including pharmacies and other medicine suppliers – and referring to staff, equipment, *and* medicines) that serve your community? What, if anything, do you think could be improved?
3) How are the costs of healthcare covered by people in this community? Which are the ways of obtaining medicines, and do you have to pay for the medicines yourself? *[Probes: health insurance, charity, out-of-pocket, other?]*

The medicines

4) Do you think that the medicines available for people in this community are usually of good quality? Have you heard of any bad experiences that people have had with medicines that were caused because they were not good quality? Details.
5) Do you know what antibiotics are, and what sort of conditions they treat? Can you give any examples of antibiotics that are available in this community?
6) *Now display the second showcard with photos of 5 different antibiotic pills that are commonly available in the study area and confirm that these are in fact antibiotics. For each photo ask*: Do people in this community use this antibiotic? What condition/illness is it used for? Is it effective or not? Do people have a preference for an antibiotic for certain conditions? If so, based on what? Why? Other details.
7) Do you think that antibiotics have any particular benefits or risks? *[Probes: side effects, antibiotic resistance].*
8) Under what circumstances do people in this community take antibiotics without a prescription or a without a recommendation from a health care worker?
9) What do you think could be done to ensure the safe use of medicines in this community?
10) Is there any sort of advertising here for medicines, and in particular for antibiotics? If yes, how far do you think this advertising influences people's choices about the medicines they buy?
11) In general, from where do people in this community purchase antibiotics?

The suppliers/seller of medicines

12) Do people usually receive instructions (verbal or written) from the supplier for using the medicines they buy?
13) In general, do you think that suppliers are well informed about the medicines they sell, so that people trust their word and want to follow their instructions? Details. [*Probe specifically for antibiotics*.]
14) Do people ever buy less medicine / fewer pills than recommended by their healthcare provider? IF so, why does this happen? Details. [*Probe specifically for antibiotics*.]

Antibiotic resistance

15) VIGNETTE: A person living in this community had a certain health condition. He went to the hospital and was given some the most appropriate medication for his condition. He took the full course of the medicine but he did not feel better. A few days later he went to another hospital for the same condition and he was given the same medicine that he was given at the first hospital. Once again, he took the full course of the medicine as the doctor instructed and still did not feel better.
    a. What do you call this condition where you take medicines for a condition but the condition does not go away?
    b. Why do you think the medication is not working?
    c. What do you think the person should do?
    d. How do you think the person can be helped?
    e. If someone takes antibiotics for a condition and it does not work, what do you call that condition?
16) What do people do with unfinished doses of antibiotics? Details (how and why).
17) Do people sometimes stop taking their antibiotics before they have finished all the pills they obtained? Details (how and why). *[Probes: do people save them? Do people ever give unused doses to other people who have a similar illness?]*
18) (a)Have you ever heard of antibiotic resistance? Do you know what it is, how it's caused, and what its implications are? [*Question to be adapted according to responses to previous questions, in particular Number 5*.] Is there anything on this topic that you would like to know more about? Details.

    (b) Have you ever heard of drugs that sometimes do not work? [Probe for examples of experience] why is that? Can this happen with antibiotics?
19) In general, where and how do people in this community learn about medicines? What do you think would be the best way to inform people in your community about proper antibiotic use and the dangers of antibiotic resistance?

Now that we have finished the discussion, I will tell the review to the group, to make sure we didn't miss anything.
Is this an accurate summary? Did we miss anything? Thank you for your participation.
Remember applause!

If you are interested, you may take along with you this leaflet which contains more information about antibiotics and antibiotic resistance. NOTE TO MODERATOR: *Provide printed information in local language.*
