# Supplementary material for: Community knowledge and practices regarding antibiotic use in rural Mozambique: where is the starting point for prevention of antibiotic resistance?
Source: BMC Public Health. 2020 Jul 29;20:1183. doi: 10.1186/s12889-020-09243-x (PMC7389384; doi:10.1186/s12889-020-09243-x)
Supplement: Supplementary file 4 — Additional file 4. [file 12889_2020_9243_MOESM4_ESM.pdf]

cism centro de investigação em saúde de manhiça 20 anos	**Appendix J. Informed consent_community member_in-depth interview**<br>"Antibiotic access and use in low- and middle-income countries" Acronym: ABACUS.	INDEPTH Network Better Health Information for Better Health Policy

The Manhiça Health Research Centre (CISM) is conducting a study on "Access and Use of Antibiotics in Communities in Low and Middle Income Countries in Asia and Africa". In Mozambique the study will take place in the district of Manhiça. We are inviting Mr./Mrs to participate in the study and share his experience in order to contribute to a better understanding of antibiotic access and use in the country.

Purpose

The purpose of this study is to learn about how people have access to and know how to use antibiotics. The ABACUS study will give us a picture of the current situation and will also tell us how to improve the availability and use of medicines in your community.

Please feel free to ask questions if anything comes up that is unclear or needs additional information related to the study.

Why was I selected?

We invite you because you are a representative member of the community we intend to study. We feel that your experience will be useful to understand more about access and use of antibiotics in your community.

What will happen in this study?

If you are interested in participating in this study, we will ask an interview to share your experience with obtaining and using medicines. We would like to know more about where antibiotics are purchased, how they are used and for what reasons. The interviews will be recorded and can last about 60 minutes. If you do not agree to record the interview, then we will take notes. The interview will be held at a time and place that offers sufficient privacy and will be agreed by you and the researcher.

What are the risks and benefits?

Your information will be kept strictly confidential and will not be shared with people outside the research group. Audio recordings will be stored securely and you will not be identified in any publication. Although there are no direct benefits, your participation will help us to formulate recommendations on what needs to be done to improve the use of medicines in the country.

What will happen with the results of this study?

We anticipate that the research will help to improve the supply and use of antibiotics in the future. These results will be shared with authorities that make decisions regarding the purchase, distribution and availability of antibiotics in Mozambique and other parts of the world.

Who should I contact for questions about the study?

If you need more information, you may contact the principal investigator of the study through the contact available below.

Principal Investigator: Khátia Munguambe, landline +258 21 810002, mobile +258 82 7356566.

CIBS: Sofia Mandjate, número fixo +258 21 810002, número de telefone celular: 82 3044440.

CNBS: Cristina Chissico, mobile phone number: 824066351

Informed consent form for the participant

The information letter has been read out loud to me or I have read the information letter and understood.

I have been given the opportunity to ask questions, and received satisfactory answers.

I understand that this study involves a focus group discussion that will be audio recorded.

I understand that the results of the study will be for scientific and public health purposes.

I understand that my participation is voluntary, and that I can withdraw my consent at any time without further explanation, and without any effect on my healthcare.

I also understand that all information collected will be treated confidentially and I freely agree to participate in this study.

After signing below, I will receive a copy of the information sheet and copy of this consent form.

I understand that there is no direct benefit for me from being in the study.

I agree with the recording of the interview. Yes |__| No |__|

Do you agree to take part in the study Yes |__| No |__|

Name of participant

|__||__||__||__||__||__||__||__||__||__||__||__||__||__||__||__||__|

Signature or fingerprints (right thumb) of the participant:

………………………………………………………………………………………………….

Signature of the Research assistant:

Right thumb

………………………………………………………………………………………

Signature of the Research assistance: |__||__||__| Date:………/……/………..
